# Degree of Alarm Fatigue and Mental Workload of Hospital Nurses in Intensive Care Units

**DOI:** 10.3390/nursrep13030083

**Published:** 2023-07-07

**Authors:** Yoonhee Seok, Yoomi Cho, Nayoung Kim, Eunyoung E. Suh

**Affiliations:** 1 Department of Nursing, Kyungil University, Gyeongsan 38428, Republic of Korea; uri303@kiu.ac.kr; 2 College of Nursing, Seoul National University, Seoul 03080, Republic of Korea; 3 Center for Human-Caring Nurse Leaders for the Future by Brain Korea 21 (BK 21) Four Project, Research Institute of Nursing Science, College of Nursing, Seoul National University, Seoul 03080, Republic of Korea

**Keywords:** alarm fatigue, mental workload, intensive care units, patient safety, nursing

## Abstract

This study aimed to determine the degree of alarm fatigue and mental workload of ICU nurses, and to clarify the relationship between these two variables. A cross-sectional, descriptive research design was used. Data were collected from 90 nurses working in four ICUs in Seoul, Republic of Korea, using a questionnaire determining their degree of alarm fatigue and mental workload. Data were collected from 6 March to 26 April 2021 and were analyzed using a *t*-test, ANOVA, and Pearson’s correlation coefficient. The average alarm-fatigue score was 28.59 out of 44. The item with the highest score was “I often hear a certain amount of noise in the ward”, with a score of 3.59 out of 4. The average of the mental workload scores was 75.21 out of 100. The highest mental workload item was effort, which scored 78.72 out of 100. No significant correlation was found between alarm fatigue and mental workload. Although nurses were consistently exposed to alarm fatigue, this was not directly related to their mental workloads, perhaps owing to their professional consciousness as they strived to accomplish tasks despite alarm fatigue. However, since alarm fatigue can affect efficiency, investigations to reduce it and develop appropriate guidelines are necessary. This study was not registered.

## 1. Introduction

The alarm sound generated by a medical device was listed as one of the top 10 health technology hazards in a report by the Emergency Treatment Research Institute in 2020 [1], with alarm fatigue being deemed a predominant medical-device-related technology risk in 2012. It is recognized as a national problem in many countries, and numerous studies are conducted on this topic annually [2]. The alarm was designed to monitor the patient’s condition in real time and to rapidly identify and manage the patient if it is out of the appropriate range [3]. The intensive care units (ICUs) of hospitals are well-equipped with a range of medical devices to extensively observe and monitor critically ill patients, and high noise and alarm sounds are induced by such devices [4]. ICU nurses are the key personnel who manage various monitoring medical devices and respond to the alarm sounds. Thus, they are the ones most directly affected by medical-device alarm sounds.

Alarm fatigue is defined as the sensory overload and desensitization that make nurses unable to respond to real threats. [5]. An inadequate situational awareness of alarm sounds is one of the main factors causing safety accidents, which can lead to serious risks [6]. In ICUs, 72–99% of the alarm sounds generated are false-positive alarms. Excessive exposure to false-positive alarms that occur without accurate physiological data violations causes sensory overload in nurses, lowering their sensitivity to alarms. Therefore, they may fail to check these alarms, putting their patients at risk [7]. When these unnecessary alarms go off, if they are not resolved correctly, the cognitive response of the brain decreases and the alarm is ignored [8]. When a patient’s crisis-related alarm is repeatedly rung, nurses’ mental workload increases as a stress response, resulting in decreased situational awareness and job performance [9]. Inadequate measures against such alarm fatigue may cause medical personnel to become insensitive to the alerts, rendering them unable to differentiate between false-positive and actual alarms in real life-threatening situations. This may further lead to their inability to recognize alarms as serious and requiring immediate action [10]. Alarm fatigue is acknowledged as a contributor to staffs’ environmental distractions and interferes with the ability of staff to perform critical patient care responsibilities resulting in patient safety issues. The Joint Commission (TJC) reported 98 alarm-related sentinel events between 2009 and 2012, of which 80 resulted in death, 13 in permanent loss of function, and five in unexpected prolonged care conditions. Of these incidences, the majority were associated with alarm malfunction, alarm misuse, or inadequate alarm settings, leading to the most common contributing factor—alarm fatigue [11].

Several studies demonstrated that continuous alarm fatigue can cause stress in nurses, with mental workload reported to be an additional cause [12]. Some studies reported that alarm-fatigue-related situations do not affect performance [9]. However, increased mental load owing to alarm fatigue affects prefrontal activity, which reduces coping strategies and increases psychophysiological demand [13]. It is thus important to clarify the degree of alarm fatigue and mental workload experienced by nurses to guarantee the quality and safety of patient care [14].

Globally, studies are actively being conducted on the effects of excessive alarm sounds on employees, nurses’ responses to alarm sounds, technology to reduce false alarm sounds, and innovations to improve alarm systems [5]. However, in Republic of Korea, there are only four studies on the topic so far. One study addressed alarm-fatigue perception, management performance, and alarm-fatigue interference factors, while another verified the effectiveness of alarm-management education [15,16,17,18]. Studies on the mental workload that affects work performance are still lacking. In addition, previous studies show a limitation in accurately measuring the alarm fatigue of nurses by using the alarm-fatigue tool developed for industrial workers. Thus, it is impossible to be completely sure of the tool’s sensitivity to alarm fatigue. Therefore, in this study, the alarm-fatigue measurement tool developed for nurses was used to understand their alarm fatigue in the ICU. This study aimed to investigate the relationship between alarm fatigue and nurses’ mental workload to provide basic data for patient safety management.

### Noise, Mental Workload, and Inhibitory Mechanism

Mental workload has a long association with human-factors research into safety-critical performance [19]. The prefrontal cortex (PFC) is a brain structure often identified as the neurophysiological source of limited resources. The PFC serves a control function during routine cognitive operations, such as action selection, retrieval/updating in working memory, monitoring, and inhibition [13]. In stressful situations, such as noise (alarm), the brain uses inhibitory mechanisms. Inhibitory mechanisms reduce neural activity associated with work or stimulation of high-level cortical areas (prefrontal cortex) to alleviate the activation of distracting neural assemblies. As a result, inhibitory mechanisms reduce the brain’s ability to take into account new information or cope [20]. Excessive and repeated alarms result in noise-induced hearing impairment, interfere with speech interpretation, negatively impact psychophysiological and mental health, diminish overall performance, and interfere with intended activities [21].

## 2. Materials and Methods

### 2.1. Design, Setting, and Participants

This study used a cross-sectional, descriptive research design to not only understand the degree of alarm fatigue and mental workload experienced by ICU nurses but also to identify the relationship between these variables. Participants were nurses working in the ICU of Seoul National University Hospital in Republic of Korea. The nurses voluntarily agreed to participate in the study based on their understanding of the study’s purpose. The study was conducted in four ICUs belonging to one hospital. Nurses in the general ward, delivery room, recovery room, and operating room were excluded from the study because the frequency of exposure to the alarm sound differed significantly from that of the ICU nurses.

### 2.2. Target Number of Participants and Calculation Basis

The G*Power 3.1.9.4 program was used to calculate the number of samples required for the study, with a significance level of 0.05, the median effect size of 0.03, and power of 80%. The correlation between alarm fatigue and mental workload was calculated and the appropriate sample size involved 82 persons. Therefore, 90 Google form questionnaires were distributed, allowing for a 10% attrition rate. However, all 90 questionnaires were returned.

### 2.3. Instruments

#### 2.3.1. Alarm Fatigue

The tool for alarm-fatigue measurement in ICU nurses was developed by Torabizadeh et al. [14]. This tool was approved for use and translation by the original author. It comprises 13 questions, rated on a five-point Likert scale ranging from 0 (“absolutely not”) to 4 (“always”). Items 1 and 9 are reverse-scored. Possible scores range from 8–44, with higher scores indicating greater influence on nurses’ performance owing to alarm fatigue. For example, in item 1, if the nurse selects “never,” it means that he/she never readjusts the limits of alarms based on the clinical symptoms of patients and always sets them in a routine range, which is wrong, so it will be scored 4 meaning a greater impact of alarm fatigue on his/her performance. At the time of development, the reliability of these 13 questions was confirmed with a Cronbach’s alpha of 0.91. The Cronbach’s alpha in this study was 0.66.

The translation was performed in three steps. First, the English tool was translated into Korean by two researchers. Next, the translated Korean tool was retranslated into English by two professional translators, who are proficient in both English and Korean. Finally, a professor who majored in nursing was commissioned to evaluate the structural similarity of the language and meaning between the original text and reverse translation.

#### 2.3.2. National Aeronautics and Space Administration Task Load Index (NASA-TLX)

The NASA-TLX is a subjective job difficulty assessment tool that was developed by NASA in the early 1980s. It is an openly accessible tool that can be used without a separate approval process [22]. The most effective way to evaluate a job’s difficulty level is to directly assess the workers with relevant experience in that job. Various survey methods have thus been developed to evaluate the cognitive load of jobs. The NASA-TLX and subjective workload assessment technique (SWAT) are well-known assessments [23]. However, the NASA-TLX has been recognized as the most stable subjective task difficulty evaluation method [24]. It can quantify the overall workload based on the average score of six questionnaire items—mental demand, physical demand, temporal demand, effort, performance, and frustration level—with scores ranging between 0 and 100 in five-point increments. The higher the score, the higher the mental workload. NASA-TLX, both the split-half reliability and Cronbach’s alpha coefficient were more than 0.80 [25]. The Cronbach’s alpha in this study was 0.80. 

### 2.4. Data Collection

Data were collected from nurses working in the ICU of a tertiary general hospital in Seoul from 6 March to 26 April 2021. Nurses who expressed their intention to participate in the study received an explanation about its purpose and procedure; written consent was then obtained from the nurses who wished to participate. Participants were then sent a survey questionnaire via email or Kakao Talk, a Korean chat app. The time required to complete the questionnaire was 10–15 min. There were no missing data in the responses among the 90 participants; thus, 90 questionnaires were used for statistical analysis.

### 2.5. Ethical Considerations

This study was approved by the Research Ethics Deliberation Committee of S Hospital for the conduct of research to ethically protect the study participants in the planning stage (no. 2102-158-1199; 22 March 2021). After obtaining consent from the hospital nursing department for data collection, the researcher directly posted an announcement of the study on the bulletin board of each department. The researcher personally interviewed the nurses who made contact after reading the notice. After asking and answering questions, if they agreed to participate in the study, a written consent form was provided to them. The nurses were assured that all data would be used for research purposes only, confidentiality would be maintained, and they could withdraw their participation at any time during the study. After data collection, a predetermined gift (mobile coupon of USD 3.0) was provided to all participants.

### 2.6. Data Analysis

Data were analyzed using SPSS 25.0 (IBM, Armonk, NY, USA). The distribution of participants’ general characteristics was analyzed based on frequency and percentage. The degree of alarm fatigue and mental workload in ICU nurses was evaluated based on the mean and standard deviation. Differences in alarm fatigue and mental workload related to the general characteristics of nurses were assessed using *t*-tests and a one-way analysis of variance (ANOVA). The relationship between alarm fatigue and the mental workload of nurses was tested using Pearson’s correlation coefficient.

## 3. Results

### 3.1. Participants’ General Characteristics

The participants’ mean age was 30.46 (5.30) years, with most participants being in their 20s (56.7%). There were 81 (90%) women, and 81 (90%) nurses with a bachelor’s degree educational qualification. Of the participants, 83 (92.2%) were registered nurses, while 7 (7.8%) were charged nurses. Of the participants, 37 (41.1%) worked in the medical intensive care unit (MICU). Nurses’ total ICU experience was 5.02 (4.95) years, with 52 (57.8%) having 1–5 years of experience. Meanwhile, 76 (84.4%) nurses stated that they oversaw care for two patients while on duty. Sixty-five (72.2%) nurses received education on alarm-sound management. Regarding ICU medical-device training within the past two years, 80 participants (88.9%) received training on the defibrillator the most, followed by 63 (70%) on the infusion pump, and 60 (66.7%) each for ventilators and physiological monitors, respectively. The number of nurses who did not experience an error related to the alarm sound was low at 16 (17.8%). Moreover, the number of nurses with direct or indirect experience of alarm-related event errors was high at 74 (82.2%). Finally, when asked whether they knew about the concept of alarm fatigue, 60 nurses (66.7%) answered that they did not (Table 1).

### 3.2. Nurses’ Alarm Fatigue and Mental Workload 

The average alarm-fatigue score was 28.59 (5.79) out of 44. The item with the highest score was “I often hear a certain amount of noise in the ward,” with a score of 3.59 (0.58) out of 4. The item with the lowest score was “I regularly readjust the limits of alarms based on the clinical symptoms of patients,” with a score of 1.05 (0.89) points. The average of the mental workload scores was 75.21 (14.70) out of 100. The performance and frustration level scores were low at 70.89 (21.58) and 70.50 (22.22) points, respectively. The highest mental load item was effort, with 78.72 (20.16) points (Table 2).

### 3.3. Alarm Fatigue and Mental Workload According to General Characteristics

A *t*-test and one-way ANOVA were performed to verify the differences between nurses’ general characteristics and alarm fatigue. Alarm fatigue showed no significant difference according to the nurses’ age, sex, education level, position, ICU type, total ICU experience, number of patients overseen per person, presence or absence of alarm sound management training, or experience of safety accidents related to alarm sounds (Table 3). 

However, alarm fatigue was significantly higher among those who were aware (vs. not) of its concept (t = 2.438, *p* = 0.017). The difference in the degree of mental workload according to general characteristics was also tested. The mental workload was higher when the number of patients cared for per person was >3, versus when there were 2 (t = 2.001, *p* = 0.048; Table 3).

### 3.4. The Relationship between Alarm Fatigue and Mental Workload

There was no significant correlation between alarm fatigue and the mental workload of ICU nurses (*r* = 0.127, *p* = 0.232; Table 4).

## 4. Discussion

This study was conducted to identify the relationship between alarm fatigue and the mental workload of ICU nurses. In the detailed item evaluating alarm fatigue, nurses reported that they experienced a significant amount of alarm noise in their working environment and that this noise was mostly generated by medical monitoring devices. The items of the alarm-fatigue questionnaire included, “I regularly readjust the limits of alarms based on the clinical symptoms of patients” and “I react differently to the low-volume (yellow) and high-volume (red) alarms of the ventilator.” This question should indicate a high score when the alarm-fatigue score is high. The participants in this study had a high overall alarm-fatigue score; however, they showed low scores for these two items. That is, if the alarm-fatigue score is high, nurses may not immediately respond to the alarm. Conversely, this study’s participants reported that they responded immediately. This finding contradicts a study showing that 81% of nurses had a delayed response to the alarm sound or deactivated the alarm sound when they experienced alarm fatigue [26]. However, this is consistent with the current finding that alarm fatigue interferes with nurses’ performance but does not affect response time [12,18]. These results show that the nurses have a sense of responsibility and alertness toward the patient. In fact, many nurses in Korea often sound an alarm when the patient’s condition is constantly unstable around the target value but nurses tolerate the alarm sound and respond sensitively to changes in the patient’s condition. Therefore, alarm fatigue may not directly affect job performance because the nurses remained alert to the alarm sound through previous patient safety accident experiences. On the other hand, we cannot rule out a bias in which the items in the survey lead nurses to choose the correct one as the type of question about moral conduct that they feel. This is because, in Korean hospital alarm management, nurses have to adjust the range of alarm sounds according to the patient’s symptoms and respond according to the red and yellow alarm sounds [15].

In addition, previous studies showed that higher alarm fatigue caused stress and increased the mental workload among nurses but there was no significant correlation in this study [9,18].

In this study, the number of nurses who directly or indirectly experienced an alarm-related event error accounted for 82.2% of the total participants, which was higher than that of 66.7% in a previous study targeting tertiary hospitals [27]. It is, therefore, necessary to investigate the causes and influencing factors of medical-device-related patient safety accidents in the future. Moreover, it is necessary to establish strategies annually to reduce alarm fatigue by analyzing technology-related factors regarding patient safety at the organizational level.

Previous difficulties related to alarm fatigue that were experienced by nurses included the absence of documented data on alarm settings (75.8%) and a lack of education concerning these settings (47.8%) [28]. In this study, more nurses (72.2%) had received training on alarm management as compared to a previous study (50.3%) [29]. However, in this study, within the previous two years, less than 70% of nurses had received training on medical devices, such as ventilators, physiological monitors, and infusion pumps, and only 80% had received training on using a defibrillator. This suggests that medical-device education occurs only once and continuous and periodic education is necessary for alarm management in the field. In addition, it was confirmed that nurses with less than three years of experience lacked knowledge regarding how to control the alarm management according to the patient’s situation and received unstructured and irregular education on alarm adjustment from the senior nurses when the alarm went off [15]. This suggests that it is necessary to develop a protocol for situational alarm management, in addition to providing training and a manual for alarm settings. In previous studies, it was found that improving education, customization of the alarm-sound parameters, and existing threshold algorithm improvement strategies were effective in reducing false positives to improve alarm fatigue [30,31].

There was no difference in alarm fatigue and mental workload between junior and senior nurses. Rather, there was a difference in nurses’ mental workload according to the number of patients per nurse. Concerning senior nurses, their workload was immense owing to the task of managing the causes of alarms, which junior nurses could not resolve. For junior nurses, it was difficult to determine which alarms were associated with which patient and what problem caused them. When the number of managed patients is small, the number of alarm sounds to be managed also decreases; thus, it is suggested that there is a difference in the mental workload for alarms according to the number of patients [12,13].

Alarm fatigue is a nursing intervention item that requires continuous management and monitoring. In this study, alert fatigue did not affect the mental workload of nurses. Since alarm-sound fatigue is at a high level, it is a factor that affects patient safety; therefore, further research is needed in the future. Efforts to effectively manage medical-device alarms and reduce alarm fatigue can prevent potentially hazardous events [32].

### Limitations

To determine the effect of alarm fatigue and mental workload, it would be more appropriate to record or observe nurses’ reactions at the time the alarm sounds. This can be considered a supplement to this study. The Cronbach’s alpha of the alarm-fatigue questionnaire used in this study was 0.66, which was a low value (more than an acceptable value of 0.7). This is in contradiction to the value of 0.91 obtained in the developed alarm-fatigue questionnaire. This is considered to be related to Korean culture or the hospital environment in Korea. In particular, in the case of questions 1 and 9, the alarm should never be turned off according to hospital rules and it should act according to the order of the alarm. In addition, in the case of Korea, they respond more sensitively to monitor alarms for a quiet environment during visits and to explain and focus more on the caregiver. To clarify this tool’s reliability, it is necessary to repeat the study with different target hospitals and target nurses. We propose to identify the reality of alarm fatigue by applying a mixed method with the addition of a qualitative method for alarm fatigue. It also suggests the consideration of alert fatigue as a health risk for nurses.

## 5. Conclusions

A nurse’s work environment directly or indirectly affects the safety and wellbeing of patients. Therefore, improving the working environment of nurses is directly related to patient safety and wellbeing. Alarm management is a nursing intervention that requires strategies to manage alarm fatigue while ensuring the delivery of safe patient care. This study was conducted to identify the degree of alarm fatigue and mental workload among nurses regarding the safety of patients and to investigate the relationship between them. However, there was no direct correlation between alarm fatigue and mental workload. This contradicts previous studies, which showed that alarm sounds cause stress and an increased mental workload among nurses. However, medical devices used in ICUs are continuously increasing and louder alarms are implemented in a noisier environment, causing psychological problems for nurses. Thus, a hospital-level alarm sound protocol and research on the reduction of alarm fatigue are necessary.

## Figures and Tables

**Table 1:** Participants’ General Characteristics (*n* = 90).

Characteristics	Category	*n* (%)	Mean (SD)
Age(years)	24~29	51 (56.7)	30.46 (5.30)
	30~49	39 (43.3)	
Gender	Male	9 (10.0)	
	Female	81 (90.0)	
Education	BSN	81 (90.0)	
	MSN	9 (10.0)	
Position	RN	83 (92.2)	
	CN	7 (7.8)	
ICU type	SICU	23 (25.6)	
	MICU	37 (41.1)	
	CCU	12 (13.3)	
	CPICU	18 (20.0)	
Total clinical experience (years)	1~5 5~10 >10	51 (56.7) 18 (20.0) 21 (23.3)	5.52 (5.39)
ICU clinical experience (years)	1~5	52 (57.8)	5.02 (4.95)
	5~10	20 (22.2)	
	>10	18 (20.0)	
Number of patients	2 patients	76 (84.4)	
	>3 patients	14 (15.6)	
Alarm-management education	Yes	65 (72.2)	
	No	25 (27.8)	
Trained medical equipment (within 2 years) (Multiple answers)	Ventilator	60 (66.7)	
	physiological monitor	60 (66.7)	
	Infusion pump	63 (70.0)	
	Defibrillator	80 (88.9)	
	Others (ECMO, CRRT, IABP, EV100, NO gas, servo-I, Masimo)	19 (21.1)	
Experience of alarm related accident	Yes	26 (28.9)	
	Indirected	48 (53.3)	
	No	16 (17.8)	
Insight of Concept	Yes	30 (33.3)	
	No	60 (66.7)	

SD, standard deviation; BSN, bachelor science of nursing; MSN, master science of nursing; RN, registered nurse; CN, charged nurse; SICU, surgical intensive care unit; MICU, medical intensive care unit; CCU, coronary care unit; CPICU, cardiopulmonary intensive care unit; ECMO, extracorporeal membrane oxygenation; CCRT, continuous renal replacement therapy; IABP, intra-aortic balloon pump; NO gas, nitric oxide gas.

**Table 2:** Nurse’ Alarm Fatigue and Mental Workload (*n* = 90).

Domain	Possible Range	Min~Max	Mean (SD)
Alarm Fatigue	8~44	11~39	28.59 (5.79)
1. I regularly readjust the limits of alarms based on the clinical symptoms of patients			1.05 (0.89)
2. I turn off the alarms at the beginning of every shift.			1.48 (1.37)
3. Generally, I hear a certain amount of noise in the ward.			3.59 (0.58)
4. I believe much of the noise in the ward is from the alarms of the monitoring equipment			3.25 (0.63)
5. I pay more attention to the alarms in certain shifts			2.42 (1.21)
6. In some shifts the heavy workload in the ward prevents my quick response to alarms			2.88 (0.89)
7. When alarms go off repeatedly, I become indifferent to them.			1.80 (1.10)
8. Alarm sounds make me nervous.			3.38 (0.70)
9. I react differently to the low-volume (yellow) and high-volume (red)alarms of the ventilator.			1.11 (0.99)
10. When I’m upset and nervous, I’m more responsive to alarm sounds			2.15 (1.19)
11. When alarms go off repeatedly and continuously, I lose my patience.			2.43 (1.10)
12. Alarm sounds prevent me from focusing on my professional duties.			2.64 (0.97)
13. At visiting hours, I pay less attention to the alarms of the equipment.			1.15 (0.97)
Mental Workload	0~100	5~100	75.21 (14.70)
Mental demand	0~100	25~100	76.78 (20.00)
Physical demand	0~100	25~100	76.39 (20.00)
Temporal demand	0~100	25~100	78.00 (20.39)
Performance	0~100	20~100	70.89 (21.58)
Effort	0~100	25~100	78.72 (20.16)
Frustration level	0~100	10~100	70.50 (22.22)

SD, standard deviation.

**Table 3:** Alarm Fatigue and Mental Workload according to General Characteristics (*n* = 90).

Characteristics	Category	Alarm Fatigue			Mental Workload		
		Mean (SD)	t/F	*p*	Mean (SD)	t/F	*p*
Age(years)	24–29	28.53 (6.32)	−0.935	0.352	74.08 (12.29)	−0.612	0.542
	30–49	29.69 (5.15)			76.30 (17.31)		
Gender	Male	29.22 (7.41)	0.102	0.919	72.59 (14.92)	−0.522	0.603
	Female	29.01 (5.70)			75.29 (14.69)		
Education	BSN	28.73 (5.82)	−1.496	0.138	75.43 (14.82)	0.803	0.424
	MSN	31.78 (5.57)			72.28 (13.23)		
Position	RN	28.98 (5.93)	−3.200	0.750	75.44 (14.39)	0.946	0.347
	CN	29.71 (4.96)			69.99 (17.89)		
ICU type	SICU	28.91 (5.17)	2.551	0.061	73.84 (17.72)	1.092	0.357
	MICU	30.43 (5.61)			73.17 (13.42)		
	CCU	29.58 (5.88)			81.67 (14.27)		
	CPICU	25.94 (6.36)			75.88 (12.78)		
Total ICU clinical Experience (years)	1~5	28.73 (6.14)	0.169	0.845	75.30 (12.70)	2.298	0.107
	5~10	29.55 (5.84)			70.32 (19.84)		
	>10	29.33 (5.17)			80.42 (13.75)		
Number of patients	2 patients	28.93 (5.92)	−0.373	0.710	73.90 (14.67)	−2.001	0.048 *
	>3 patients	29.57 (5.58)			82.32 (13.14)		
Alarm management education	Yes	28.68 (6.04)	−0.933	0.354	74.41 (15.18)	−0.627	0.522
	No	29.96 (5.30)			76.59 (13.34)		
Experience of alarm related accident	Yes	30.23 (6.46)	1.810	0.170	75.68 (14.41)	0.096	0.909
	No	26.75 (5.53)			73.64 (19.29)		
	Heard that	29.14 (5.48)			75.12 (13.29)		
Insight of Concept	Yes	31.10 (5.09)	2.438	0.017 *	73.91 (13.75)	0.504	0.615
	No	28.00 (5.96)			75.57 (15.17)		

SD, standard deviation; BSN, bachelor science of nursing; MSN, master science of nursing; RN, registered nurse; CN, charged nurse; SICU, surgical intensive care unit; MICU, medical intensive care unit; CCU, coronary care unit; CPICU, cardiopulmonary intensive care unit; ECMO, extracorporeal membrane oxygenation; CCRT, continuous renal replacement therapy; IABP, intra-aortic balloon pump; NO gas, nitric oxide gas. * *p* < 0.05

**Table 4:** The Relationship between Alarm Fatigue and Mental Workload (*n* = 90).

	Alarm Fatigue	Mental Workload
Alarm Fatigue	1	0.127 (0.232)
Mental Workload	0.127 (0.232)	1

## Data Availability

Not applicable.

## Public Involvement Statement

No public involvement in any aspect of this research.

## Guidelines and Standards Statement

This manuscript was drafted against the (STROBE) for a cross-sectional study, descriptive research.
